# Willingness to Pay for Hexanal Technology among Banana Farmers in Meru County, Kenya

**DOI:** 10.1155/2021/6676148

**Published:** 2021-01-29

**Authors:** Jane N. Kahwai, John I. Mburu, Martin O. Oulu, Margaret J. Hutchinson

**Affiliations:** ^1^Department of Agricultural Economics, University of Nairobi, P.O. Box 30197, Nairobi, Kenya; ^2^Department of Plant Science and Crop Protection, University of Nairobi, P.O. Box 30197, Nairobi, Kenya

## Abstract

From the perspective of food categories, fresh produce are the leading sources of food loss and waste globally. Their highly perishable nature shortens their shelf-lives leading to high postharvest losses if not properly handled. Currently, these losses are estimated at sixty-six percent based on total weight. Reduction of these losses will ensure constant supply of food along the supply chain as well as economic empowerment of the rural poor. Hexanal which is a naturally occurring compound has been developed as an intervention to prolong shelf-life of delicate tropical fruits such as bananas while also maintaining their quality. However, empirical evidence is still required on the usefulness of hexanal to farmers. It is envisaged that such evidence would inform scaling up of the technology in Kenya. This study assessed willingness to pay for hexanal and the factors influencing WTP amounts among banana farmers in Meru County, Kenya. Primary data was collected from 130 respondents who were grouped into aware and not aware of Hexanal. Results indicate that farmers who are aware of hexanal had a higher mean WTP Ksh 466.47 (US $4.66) compared to those not aware Ksh 331.86 (US $3.32). Factors such as age and income influenced the WTP amounts between subsamples. The major key policy implication of the study is the importance of stakeholders investing in the dissemination of information on hexanal among farmers to enhance uptake.

## 1. Introduction

Across the food categories, fruits and vegetables are the major causes of food loss and waste globally. According to literature, these losses are estimated at 66% based on total weight [1, 2]. Fresh produce has a very short shelf-life which predisposes them to deterioration when not adequately handled during harvesting, transporting, storage, marketing, and consumption. According to [3], postharvest loss is defined as the measurable qualitative and quantitative loss along the postharvest value chain.

These losses are higher in developing countries compared to the developed countries [4]. This is due to the lack or poor agricultural practices and specialized facilities such as cooling facilities, packaging materials, marketing systems, and infrastructure in developing countries [5] which delays produce reaching the markets on time and in good condition. In order to ensure increased availability of food along the supply chain from the existing production, it will be necessary that these losses and quality deterioration are reduced [6].

In Kenya, the fruit subsector is very important due to its tremendous contribution to the economy. In 2016, the subsector contributed Ksh 57 billion (US$ 570 million) which accounted for 27% Kenya's value of horticultural produce [7]. Bananas (*Musa* spp.) were ranked first in terms of production with 1.24 million tons being produced under 63074 Ha of land which was an increase from the 60743 Ha in 2015. The production was reported to be worth KSh 18.1 billion (US $ 180 million) accounting for 31.6% of the total fruits' production in the country [7]. The increased production has been attributed to the shift from backyard to commercial farming of bananas as a propoor agroenterprise [8, 9]. The major banana-producing regions in Kenya together with their percentage contributions are Meru (20%), Murang“a (11.7%), Kirinyaga (8.1%), Taita Taveta (6.6%), and Tharaka Nithi counties (5.6%) [10]. The most preferred banana variety currently is the Cavendish (both the Dwarf and Giant) at 23%.

The banana enterprise is highly commercialized in Kenya as farmers sell 86% of their output [11]. Commercialization of bananas especially in Central and Eastern regions can be attributed to the decline in traditional cash crops such as coffee as well as the recent success in the introduction of high yielding tissue culture which includes Grand Nian, Williams, Chinese Cavendish, and Giant and Dwarf Cavendish varieties ([12], [13]). The market for banana is also rapidly expanding due to the demand for consumption of healthy foods.

Supply of the banana fruit is however lagging behind as the subsector is faced with high-post harvest losses which are estimated at 40% [5], the main cause being poor postharvest handling practices. A ripe banana is a delicate and perishable fruit [8] with a shelf-life of only 3-4 days [14]. The high-post harvest losses reduce the availability of the fruit in the supply chain as well as farmers' incomes. This is because farmers are forced to sell their produce at farm gate prices due to glut of the fruit in the market thereby making losses. It is therefore important to extend banana shelf-life to avert more postharvest and economical losses as well as enhance food and nutritional security which is one of the UN Sustainable Development Goals (SDGs) [15]. In addition, bananas are essential for human nutrition as they are a source of vitamins (B6 complex), calories, phytonutrients, and minerals (magnesium and potassium) [16].

### 1.1. Approaches and Technologies for Reducing Postharvest Losses in Bananas

Against this background, it is important to introduce and promote nonsophisticated technologies to farmers to delay ripening and prolong the shelf-life of bananas and other perishable farm produce at ambient conditions. This will ensure farmers benefit from the increased production and high demand of the bananas in the country. The locally available banana preservation methods/technologies include sun-drying, charcoal and brick coolers, and value addition into products such as banana flour.

Hexanal (C_6_H_12_O) which is a nanotechnology formulation that is organic in nature has been developed to prolong the shelf-life of mature green fruits such as strawberries, sweet cherries, and sweet bell peppers [17, 18]. Hexanal works by inhibiting the production of ethylene, thereby delaying ripening of fruits [19]. It has been found to be effective in increasing the shelf-life of fruits. In Kenya, efficacy trials carried out between 2014 and 2018 on bananas, mangoes, and pawpaw proved hexanal to be effective when used as either a spray or a dip. Bananas sprayed with hexanal solution remained on the trees for an extra 12 to 18 days before ripening based on peel color changes while dipping fruits in the solution prolonged their shelf-life by 9 days as well as improving their quality in terms of firmness and uniformity in color during ripening [20, 21].

Hexanal is insoluble in water, and to increase its solubility, a formulation is made known as the enhanced freshness formulation (EFF) that contains Tween 20, ethanol, and distilled water. Hexanal has been shown to have no negative effects on the human body. This is because it is oxidized after 48 hours to hexanoic acid which is further oxidized to water and carbon (IV) oxide during the respiration process [22]. Hexanal is also not traceable in treated tissues after 48 hours of treatment (http:/http://www.accessdata.fda.gov/). Therefore, fresh produce treated with hexanal do not require any special handling or cause any harmful effects. Currently, hexanal is not yet available in the Kenyan market and is only in use under experimental basis. However, efficacy trials have been successful and it is expected to be approved by the Kenya Plant Health Inspectorate Service (KEPHIS) for commercial purposes. In addition, hexanal is already in use and commercialized in other countries such as Canada and India.

To ensure broad and sustained adoption of hexanal technology, adequate information should be generated that will assist the product developers as well as other stakeholders to know how much farmers are willing to pay for the technology. Adoption of hexanal technology will ensure farmers have a little more time to look for premium markets thereby reducing their losses and increasing their incomes. The objective of this study was therefore to assess how much banana farmers are willing to pay for hexanal technology and the factors influencing their WTP.

### 1.2. Theoretical Framework

During the introduction of a new product in the market, the proponents are more interested in the production costs and consumer demand of the new technology. This is because these are the main considerations in the pricing of products and adoption by consumers. Estimating production costs is never a challenge unlike assessing the consumer demands for new products whose market prices are not yet set. This necessitates the need to create a hypothetical market scenario which is similar to real markets to enable economists assess consumer demands for new products [23] as well as their perceptions.

The current study was anchored on the random utility theory. The theory is based on the hypothesis that individuals are rational decision makers whose aim is to maximize utility relative to choices available. According to the theory, an individual will always select the alternative that maximizes his or her utility. Utility assigned to each alternative is determined by several attributes or characteristics, and since an individual's direct utility cannot be measured; their choices can be observed [24]. The utility of an alternative in this case depends on the attributes of the alternative as well as the individual whereby some are observable while others are unobservable to the analyst [25]. The observed attributes are represented as explanatory variables (deterministic component) while the unobserved attributes are treated as random variables (stochastic component) in the utility function [26].

According to [27], random utility models have demonstrated their usefulness over time in guiding of innovation development. [28] noted that survey responses from CVM are economically meaningful, as they are comprised of a utility maximizing response to a survey questions hence being consistent with the utility maximization economic model. Since utility maximization is subject to a budget constraint, a consumer can only choose a good that maximizes his/her utility but not above his/her budget as his/her demand will be constrained. With measurement of a good's quality being represented by *q* a rational individual will always choose the level of market good represented by xm that maximizes their utility forming a Marshallian demand curve, xm (*p*, *y*, *q*); whereby (*p* is the current market price of the good and *y* is the individual's income). Therefore, WTP estimates are useful in agribusiness as they identify positions on the demand curve beyond which returns on investments are positive [29].

## 2. Materials and Methods

### 2.1. Study Area

**Figure 1:**
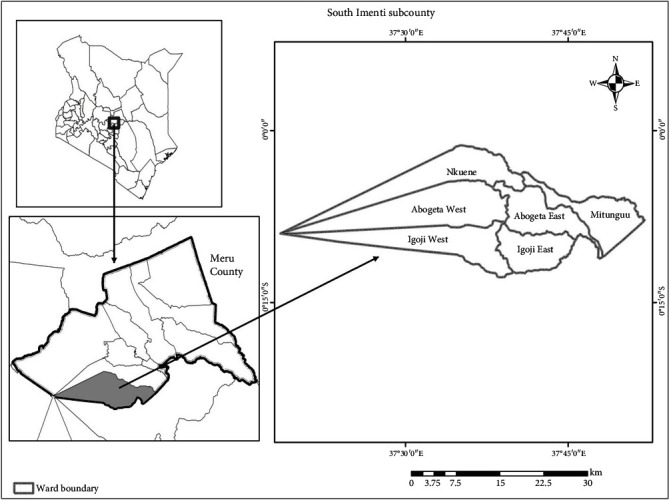
Map showing study areas in Imenti South subcounty. Source: created from Arc-GIS by Author.

Meru County (Figure 1), which is approximately 225 km northwest of Nairobi, is located on the eastern part of Mount Kenya covering an area of 6936 sq km. The county borders four other counties, namely, Laikipia to the west, Tharaka-Nithi to the southwest, Isiolo to the north, and Nyeri to the southwest. The area lies between altitudes of 300 and 5199 meters above the sea level. Climate is cool and warm with annual average temperatures ranging between 8°C in cold seasons and 32°C in hot seasons. The average annual rainfall received in the region is 1250 mm [30].

Agriculture is the main economic activity in the county with tea, coffee, and bananas being the main cash crops produced. Additionally, dairy and fish farming is also practiced mainly for local consumption. In 2014, 9715 tonnes of bananas were produced from the county which was an increase from 6884 tonnes in 2013 [30]. Tourism is also a major economic activity as the county has several tourist attraction sites such as the Lewa Conservancy, Meru Museum, Meru National Parks, and Mt. Kenya National Park. Despite the region being cosmopolitan, majority of the people are Meru speaking.

Meru County comprises of nine subcounties, and the current study was based in South Imenti subcounty. The subcounty which covers an area of 739 sq km is the most developed in Meru with a good and vast road network that facilitates transport of inputs and produce to markets. According to the 2009 household survey, population was at 179604 [31]. Furthermore, the subcounty was purposively selected as it is where efficacy trials of hexanal on bananas were conducted [21].

### 2.2. Sampling and Research Design

Data collection was conducted in April 2018 as a second follow-up survey on individuals interviewed during a baseline survey by the University of Nairobi in collaboration with the International Development Research Centre (IDRC) in 2016. Criteria for selecting respondents for the current study were therefore grounded on the respondents from the baseline survey. The study only targeted farmers who produce bananas either for commercial or subsistence purposes. A household unit was used as the sampling unit.

The baseline study used a multistage sampling procedure in selecting respondents. In the first stage, Meru County was purposively selected based on empirical evidence as the region with the highest volume of bananas in terms of production and marketing ([32], [9]). The second stage involved mapping out banana producer groups in the area from which a sampling frame comprising banana producers in South Imenti subcounty was created. A sampling frame of 1800 households producing bananas was generated from banana farmer groups and cooperatives in the region. Given the known population from which sampling was conducted, Cochran (2007) formula for known population was used to generate the sample size as shown below:

(1) $$\begin{array}{c} \frac{n_{0}}{1+\left(n_{0-1}/N\right)}=n, \end{array}$$

(2) $$\begin{array}{c} \frac{384}{1+\left(\left(384-1\right)/1800\right)}=317. \end{array}$$

From the formula, the ideal sample size for this study was 317 respondents. However, due to constraints in time and resources, systematic random sampling was used in the third stage to select every 10^th^ respondent on the list which resulted in a representative sample of 180 households. Systematic random sampling eliminates bias by guaranteeing that each household has an equal opportunity of being selected [33]. The current study however managed to interview only 130 respondents which were occasioned by dropouts during the follow-up interviews.

Interviews were only conducted with the household head, spouse, or both. Absence of either the head or spouse in a household resulted in termination of the interview, and the household was systematically substituted. Since the households were specifically selected from banana production groups, replacement was only from a specific household list which greatly reduced the sample size. The main challenges encountered during data collection was absence of respondents, a tough terrain, and bad weather which made it difficult in accessing some households as well as increasing costs.

In the current study, the respondents were categorized into two groups comprising of the treatment and control groups. The treatment group (aware) (*n* = 52) attended a dissemination workshop where they were trained on the use and benefits of hexanal in February 2018. On the other hand, the control group (not aware) (*n* = 78) comprised of farmers that did not attend the dissemination workshop and were not aware of the existence of hexanal. Enumerators familiar with the local dialect collected data on sociodemographics of the respondents, farm characteristics, infrastructure, external support services, and WTP values.

### 2.3. Data Collection

#### 2.3.1. Willingness to Pay Elicitation Format and Bidding Process

The study used contingent valuation method which is one of the stated preference (SP) approach used to elicit the maximum amounts farmers were willing to pay for hexanal technology. According to [34], CVM is the most common approach used to elicit information on the value of nonmarket goods using questionnaires. Despite its popularity, the approach harbors some concerns in relation to its associated bias hypothetical premise [35]. However, these concerns can be addressed by improving the design of the survey as well as the administration of the questionnaire [36]. There are four major elicitation techniques used in CVM, and they include the open-ended questions (OE) whereby respondents are directly asked for their maximum WTP, the payment card technique which involves a respondent picking a card containing their preferred maximum WTP value for the good in question, dichotomous choice (DC) technique whereby the respondent is asked to state ‘yes' or ‘no' to a predetermined bid that is set to reflect the maximum WTP, and the bidding game [37].

The choice of elicitation technique to use depends on the nature of good to be valued as well as the resources available for survey. In the case of this study, bidding game technique was used where the respondents were assigned a specific initial bid and were required to answer with a ‘yes' or ‘no' The difference between the dichotomous choice and the bidding game is that in the latter, the process is continuous whereby the interviewer increases the bid amount if the previous response was ‘yes' until they obtain a negative response and reduces the initial bid amount if the previous response was ‘no' up to a point a positive response is obtained and the highest amount the respondent is WTP is recorded [38].

Data was obtained on the WTP amounts for both groups of farmers who are aware as well as those not aware of the hexanal technology. Farmers who were already aware of the technology were given a brief reminder of the attributes of the technology, how to use the technology, and its' benefits as they had already attended a dissemination workshop on the same. As for the case of farmers not aware of the technology, a hypothetical scenario was provided in order enable the elicitation of the maximum WTP amount from the farmers. Information on mix ratios of Hexanal was explained of diluting 0.25 liters of hexanal with 12.5 liters of water. It was explained to them that the solution would be enough to spray 125 bunches of bananas or dip as many fruits till the solution is completely used. The hypothetical scenario was designed as follows: *“banana production supports many farmers economically in Kenya. However, lack of access to proper post-harvest handling techniques contributes to great losses of up to 40% each year. There is an organic pre-harvest dip and spray known as Hexanal technology {which is an Enhanced Freshness Formulation (EFF)} that is capable of prolonging fruit shelf life by 21days on the trees and 17* days *in storage (at room temperature) to 26* days *in cold storage. Field trials carried out in Kenya show it is very effective in prolonging shelf life in mangoes and bananas while causing no harmful effects on humans. The product is currently not available in the market but considering the costs of importation it would cost Ksh.400 (US $4) per 0.25Litres. If the product was introduced in the market and you were required to pay for it, would you be willing to pay for it? Would you be willing to pay Ksh400 per 0.25L?”*

Iterative bidding was then used to elicit the maximum WTP. First, the enumerator explained to the respondent that they would have to pay cash for the product or purchase it through credit from an agro-dealer and repay later after harvesting. A bid of ±Ksh 50 (US $0.5) was used whereby if the answer was “yes” to the initial amount of Ksh 400 an increment of the bid amount was added until the respondent said “no.” In case the respondent responded “no” to the first amount an equal decrement of the bid used until the respondent revealed the amount they are willing to pay by answering with a “yes.” The revealed amount was recorded as the maximum amount farmers are WTP. The base price of Ksh 400 of the hexanal technology was obtained from the aggregation of the components' current market value/prices used to formulate hexanal.

#### 2.3.2. Data Analysis

The mean WTP amounts and the factors likely to influence farmers' WTP were all analysed using econometric software Statistical Package for Social Scientists (SPSS) version 20, and STATA version 14. SPSS was used for data entry and cleaning while STATA was used to estimate the mean amounts farmers are willing to pay for the hexanal technology as well as their determinants. Data was analysed separately for the two groups of farmers to obtain differences in WTP amounts and their determinants between the treatment farmers and control farmers.

#### 2.3.3. Econometric Estimation

In the case of choice discrete response format as is the case in this study, it is assumed a farmer is interested in reducing postharvest losses of his fruits. Therefore, his/her corresponding indirect utility function would depend on *q* which is the novel product to be valued; *p*, prices of market goods; *z* which is the farmer's characteristics; *y* representing the farmer's income, and *ε* representing some stochastic components of preferences of the farmer which are unobservable to the researcher and hence treated as random [26]. Therefore, the farmer will be faced with the following indirect utility function *V* (*q*°, *p*, *y*, *z*, *ε*). With introduction of hexanal technology, a farmer is confronted with the opportunity of prolonging freshness of his/her fruits which will require a change from using product *q*° which is the traditional postharvest handling techniques to *q*^1^, which is the hexanal technology that has proved to be effective in prolonging the shelf-life of mangoes, bananas, and pawpaw in Kenya. Hexanal technology is more effective and has greater benefits to the farmer than traditional techniques hence *q*^1^ > *q*°. It is assumed the farmer perceives the change as an improvement in terms of incomes from the reduced losses and hence his/her indirect utility is as follows;

(3) $$\begin{array}{c} V\left(q^{1},p,y,z,\varepsilon \right)\geq V\left(q^{{}^{\circ}},p,y,z,\varepsilon \right) \end{array}$$

However, when the farmer is informed that the change would cost Ksh *A* the farmer would only be willing to pay (by replying “yes”) to the amount only if

(4) $$\begin{array}{c} V\left(q^{1},p,y-A,z,\varepsilon \right)\geq V\left(q^{{}^{\circ}},p,y,z,\varepsilon \right), \end{array}$$

and “no” otherwise, as his/her main objective is to maximize utility.

The maximum amount a farmer is willing to pay for a change from *q*° to *q*^1^ can be expressed using the compensating variation measure whereby *C* satisfies

(5) $$\begin{array}{c} V\left(p.q^{1},y-C,z,\varepsilon \right)=V\;\left(p.q^{{}^{\circ}},y,z,\varepsilon \right). \end{array}$$

Thus, *C* = *C* (*p*, *q*^1^ *q*°, *y*, *z*, *ε*) is a farmer's maximum WTP for the change. If the stated price in the bid question is lower than the above WTP, a farmer will answer “yes” and “no” otherwise and hence

(6) $$\begin{array}{c} \mathrm{Max}\text{ WTP}=C=C\left(p,q^{1}q^{{}^{\circ}},y,z,\varepsilon \right)\geq A. \end{array}$$

Adoption of the hexanal technology is perceived as a farmer's way of improving the quality and freshness of his/her fruits by changing postharvest handling techniques from *q*° to *q*^1^. Alternatively, the WTP for the change in this case is expressed as

(7) $$\begin{array}{c} \text{WTP}=\pi \left(q^{1},p,w\right)-\left(q^{{}^{\circ}},p,w\right), \end{array}$$

whereby *w* is the vector of input prices and *p* is the vector of output prices, which yields the following indirect restricted profit function *π* (*p*, *w*, *q*). In reference to equation (7) above, WTP is the amount of profit the farmer would be ready to forego to obtain the hexanal technology *q*^1^ rather than using traditional techniques *q*°. The farmer is likely to adopt the novel product which is the hexanal technology if he/she perceives it to provide higher utility. WTP in this case was evaluated using averaging the ‘Yes' individual bid responses which resulted in the mean amount WTP in Ksh.

A two-limit tobit model was used to assess the factors influencing willingness to pay for hexanal with logWTP as the dependent variable. Tobit model was found to be superior to OLS and probit models due to the nature of the dependent variable which was scaled between 2 and 3. WTP was censored from above and below due to the presence of outliers within the data. Tobit model uses the maximum likelihood estimation that directly estimates *σ* and *β*˜.

Theoretically, the model is presented as follows [39]:

(8) $$\begin{array}{c} Y^{*}=X\beta +\varepsilon , \end{array}$$

where *Y*^∗^ is the latent (hidden) variable that is unobservable, *β* is the vector for some unknown coefficients, and *X* is the vector for independent variables while *ε* is the error term which is assumed to be independently distributed with a mean of zero and a variance of *σ*^2^.

Two similar regressions for both the treatment and control groups were run using identical sets of independent variables. The estimating equations are as follows:

(9) $$\begin{array}{c} \text{WTP}\left(Y^{*}\text{treatment}\right)=\beta_{0}+\beta_{1}\text{AGE}+\beta_{2}\text{INC}+\beta_{3}\text{SEX}+\beta_{4}\text{EDU}+\beta_{5}\text{CRDTACC}+\beta_{6}\text{LANDSIZE}+\beta_{7}\text{GRPMBRSHP}+\beta_{8}\text{PERCACCEPT}+\beta_{9}\text{INITIALBID}+\beta_{10}\text{OCCP}+\beta_{11}\text{DISTMKT}+\varepsilon_{i}\cdots , \end{array}$$

(10) $$\begin{array}{c} \text{WTP}\left(Y^{*}\text{control}\right)=\beta_{0}+\beta_{1}\text{AGE}+\beta_{2}\text{INC}+\beta_{3}\text{SEX}+\beta_{4}\text{EDU}+\beta_{5}\text{CRDTACC}+\beta_{6}\text{LANDSIZE}+\beta_{7}\text{GRPMBRSHP}+\beta_{8}\text{PERCACCEPT}+\beta_{9}\text{INITIALBID}+\beta_{10}\text{OCCP}+\beta_{11}\text{DISTMKT}+\varepsilon_{i}\cdots . \end{array}$$

## 3. Results and Discussion

### 3.1. Sample Statistics of the Respondents Are as Shown in Table 1 below

### 3.2. Households' Willingness to Pay for Hexanal

#### 3.2.1. Estimation of Mean WTP between Categories of Farmers

The results show that both groups of farmers in Meru County are willing to pay positive amounts for the hexanal technology (Table 2). The minimum amounts farmers were willing to pay for hexanal was Ksh 100 (US $1) for both aware and not aware farmers, respectively. Farmers who were aware of hexanal had higher mean WTP of Ksh 466.47 (US $4.66) compared to Ksh 331.86 (US $3.32) for farmers not aware of the technology. The mode for both categories of farmers was Ksh 400 (US $ 4).

It was hypothesized that there would be no difference in the mean amounts farmers are willing to pay between the farmers aware and those not aware of the technology. However, results from *t*-test testing the hypothesis of equal WTP amounts was rejected at 1% level of significance (*p* < 0.01). The mean WTP amount for farmers who attended the dissemination workshop is statistically higher than for those who never attended. Specifically, farmers aware of hexanal are willing to pay Ksh 134.61 (US $1.34) more than those ones not aware of the technology. Therefore, the results are an indication that access to information on existence of a new technology increases the acceptance and WTP amounts for the technology. These results are consistent with [40] who found out that farmers who had prior knowledge about a hermetic storage bag in Kenya had a higher WTP compared to those with no prior knowledge. In addition, the mean WTP for farmers aware of the technology is also higher than the initial bid value which is an indication of undervaluation of the hexanal technology which can happen in cases where prices for nonmarket goods are set with little or no consideration for farmers' preferences [41]. For both groups of samples, the median WTP was found to be lower than the mean WTP amounts for hexanal. The findings are consistent with literature whereby [42] used the CVM approach to study the WTP for ecotourism development in Hong Kong and found out that the median WTP was 16% lower than the mean WTP.

### 3.3. Factors Influencing WTP for Hexanal

Identical sets of independent variables were used in the tobit regression model for both groups of farmers, those aware and those not aware of hexanal. Results on Table 3 below indicate that LR chi^2^ statistic for both groups were significant at 1% level of significance (*p* < 0.01) which is an indication variables included in this regression significantly contribute to the changes in the maximum amount households are willing to pay for hexanal. The pseudo-*R*^2^ was 53.15 and 24.25 for the not aware and aware groups, respectively. The values indicate that independent variables included in this model could explain 53.15% and 24.25% variation in the maximum WTP, respectively.

Among the explanatory variables, the initial bid amount positively influenced (*p* < 0.01) the maximum WTP for both groups of farmers. This is an indication that increasing the bid amount results in increased mean WTP for hexanal.

Based on the economic theory by [43], increasing the bid amounts through iterative bidding approaches, such as in this case, increases the demand for the product thereby increasing prices. The findings are an indication that households in Meru County believed the initial bid amount to be the true value of the technology and based their maximum WTP on the amount. Additionally, the findings could be indicative of the likelihood of occurrence of a starting point bias that could explain the high influence of the initial bid on the WTP amounts.

Age of the respondent negatively influenced (*p* < 0.1) the mean WTP amounts among farmers not aware of the technology. The results are an indication that older farmers not aware of the technology were willing to pay lower amounts for hexanal compared to younger farmers. These findings are consistent with [44, 45] who also found out that farmers' age negatively influenced adoption of agricultural innovations. Several studies have also found out that younger farmers are more receptive towards innovations and hence more likely to adopt new agricultural technologies compared to older farmers. Older farmers have been reported to be more conservative compared to younger farmers as well as more risk averse [46, 47].

Gender of the respondent (being male) was found to negatively influence the mean WTP amounts among households aware of hexanal (*p* < 0.01). This means the mean WTP amounts were less for male farmers compared to female farmers. According to a study by [48] on gendered analysis of banana value chain in Meru County, the research found out that women dominated the retail marketing channel. The findings explain why the WTP for hexanal is higher for women compared to men as women view hexanal as a technology capable of reducing their losses thereby increasing incomes from their sales.

The explanatory variable ‘main occupation of the respondent' negatively influenced WTP amount (*p* < 0.05) among the farmers who attended the dissemination workshop and are aware of the hexanal. Farmers who practice farming as their main occupation had reduced WTP amounts compared to farmers engaging in other nonfarm activities. This could be explained in that farmers involved in other activities viewed hexanal a solution to save time spent on farm activities and looking for markets, which increased the demand of hexanal among them.

Marital status of the respondent (being married) positively influenced (*p* < 0.05) WTP amounts among famers aware of the technology. Married farmers were more likely to pay higher amount for hexanal compared to the unmarried if they perceived hexanal capable of increasing their incomes which would enable them take better care of their families.

Distance to the market center negatively influenced the WTP amounts (*p* < 0.1) among households not aware of the technology. Living far away from market centres reduced a farmer's WTP amount. The disincentive for this group of farmers could be from lack of information on the uses and benefits of hexanal in prolonging the shelf-life of the fruit and therefore causing farmers to incur more transaction costs in search of the information leading to low demand for the technology. This variable had no significant influence on the WTP among farmers aware of hexanal.

Land size which was measured in acres positively influenced (*p* < 0.1) the WTP amounts among farmers aware of the technology. Farmers with larger farm sizes were willing to pay higher amounts for hexanal to reduce postharvest losses due to their high production of bananas. A larger land size under banana production meant increased output which required good postharvest handling to avoid losses. This led to the increased demand for the technology among farmers already aware of the benefits of hexanal [49]. Farmers with small land sizes are in most case not able to invest in expensive technologies.

The variable income (income received from banana sales) had a positive influence (*p* < 0.05) on the WTP amounts among farmers who were aware of hexanal. Households obtaining higher incomes from banana production were willing to pay higher amount for hexanal. Income can be used as a proxy for a household's ability to purchase quality farm inputs. This is consistent with the broad range of literature which shows that households with higher incomes have higher chances of being early adopters of new technologies [50]. Therefore, increasing a household's income will increase demand for inputs such as hexanal if they perceive it to reduce their losses. [45] also found out that farmers with high incomes are more likely to pay more for quality and healthier foods while [51] who studied WTP for Aflasafe in Kenya found out that households with higher incomes were willing to pay higher prices for the biopesticide in order to produce maize free from aflatoxins.

The perception on social acceptance of the technology positively influenced the maximum WTP (*p* < 0.5) among farmers not aware of the technology. Households that perceived hexanal to be a socially acceptable product which they would be able to incorporate it as one of their post-harvest management practices were willing to pay higher amounts for it. The findings concur with [52] who found out that consumers' perceptions influenced their maximum WTP for genetically modified rice.

## 4. Conclusion

The study is aimed at assessing the WTP amounts for hexanal and the factors influencing them among banana farmers in South Imenti subcounty. Overall, farmers are willing to pay positive amounts for the hexanal technology. This provides sufficient guide on the pricing mechanisms for the technology developers and the extension providers. In addition, farmers who were aware of the technology had higher WTP amounts compared to those not aware of the technology which explains the critical role of information in enhancing the acceptability/adoption of a technology.

WTP was influenced by several factors between the two groups. Factors that positively influenced WTP included initial bid amount, marital status, land size, income, and perception on social acceptability of hexanal. On the other hand, age, gender, main occupation, and distance to market center were found to negatively influence WTP amounts. Therefore, product developers should ensure pricing of the technology takes into account the households' sociodemographic characteristics that were found to influence amounts farmers are willing to pay.

Therefore, stakeholders should provide sufficient information in order to enhance perception on social acceptability of the technology as it was found to positively influence the WTP. Since distance to market center negatively influenced WTP, extension providers should educate farmers living away from town centers through farmer field days and trainings to increase awareness on hexanal in order to enhance its adoption. Farmer's age negatively influenced WTP among farmers not aware of the technology; hence, there is a need for product developers to conduct more dissemination workshops among younger farmers not aware of the technology in order to demonstrate the effectiveness of the technology.

The current study only focused on the WTP amounts and did not consider if hexanal is actually profitable for use by the banana farmers. Assessing the cost-benefit analysis of the technology is therefore a potential area of future research. Output from the cost-benefit analysis could be useful in providing more evidence for increased dissemination and commercialization of the technology as well as aiding farmers in making more informed investment decisions.

## Figures and Tables

**Table 1:** Socioeconomic characteristics of sampled households.

Variable	Min	Max	Mean (SD)
Household characteristics			
Household size	1	6	3.36 (1.36)
Age of household head in years	25	90	60.6 (14.4)
Years of farming experience	0	70	30.98 (15.67)
Years of schooling of household head	0	18	9.04 (3.6)
Annual income from banana production (Ksh)	1400	720000	121536 (115159)
Annual total household income (Ksh)	23760	1979000	333793 (279737)
Marital status of household head (1 = married, 0 = otherwise)	0	1	0.79 (0.41)
Gender of household head (1 = male, 0 = female)	0	1	0.84 (0.37)
Main occupation of household head (1 = farming, 0 = otherwise)	0	1	0.78 (0.41)
Farm characteristics			
Total land size (acres)	1	40	2.9 (4.02)
Land tenure (1 = titled, 0 = otherwise)	0	1	0.72 (0.45)
Infrastructure			
Distance to input shop (km)	0	20	0.92 (2.02)
Distance to banana collection center (km)	0	11	2.5 (2.6)
External support services			
Access to credit (1 = yes, 0 = no)	0	1	0.14 (0.35)
Group membership (1 = yes, 0 = no)	0	1	0.59 (0.49)
Years of group membership	0	50	7.47 (10.84)
Access to extension (1 = yes, 0 = no)	0	1	0.23 (0.42)
Perception on social acceptability of hexanal	-1.59	2.49	5.04 (0.99)

Source: Survey data, 2018; SD: standard deviation; Ksh: Kenyan shillings; km: kilometer. Conversion Ksh 100 = US $1.

**Table 2:** WTP estimates (Ksh per 0.25 liters of hexanal).

Household category	Valid n	Mean	SD	Min	Max	Mode	Median	*t* value
Meru (aware)	78	466.47	203.7	100	1000	400	400	
Meru (not aware)	52	331.86	126.27	100	600	400	325	
								-4.6518∗∗∗

Source: Survey data, 2018. Ksh: Kenyan shillings. Conversion Ksh 100 = US $1.

**Table 3:** Factors influencing WTP for hexanal.

Variable	Control	Treatment
LogMAXWTP (Ksh)	Coefficient (robust SE)	Coefficient (robust SE)
Initial bid amount (Ksh)	0.225 (0.035)^∗∗∗^	0.339 (0.052)^∗∗∗^
Age of household head (years)	−0.003 (0.002)^∗^	0 (0.002)
Gender of household head (1 = male, 0 = female)	0.016 (0.054)	−0.283 (0.043)^∗∗∗^
Main occupation of household head (1 = farming, 0 = otherwise)	-0.032 (0.032)	−0.146(0.05)^∗∗^
Marital status of household head (1 = married, 0 = otherwise)	0.035 (0.06)	0.089 (0.038)^∗∗^
Years of schooling of household head	-0.005 (0.006)	-0.006 (0.007)
Distance to market (km)	−0.017 (0.0090)^∗^	-0.04 (0.008)
Land size (acres)	0	0.025 (0.014)^∗^
Annual income from banana sales (log)	-0.033 (0.024)	0.014 (0.006)^∗∗^
Perception on social acceptability of hexanal	0.042 (0.017)^∗∗^	0.11 (0.019)
Group membership (1 = yes, 0 = no)	0.043 (0.042)	-0.063 (0.04)
Constant	2.99 (0.327)^∗∗∗^	2.487 (0.128)^∗∗∗^
Log pseudolikelihood	26.01	27.3
LR chi^2^ [40]	53.02^∗∗∗^	56.95^∗∗∗^
Pseudo-*R*^2^	53.15	24.25

Note: ^∗^, ^∗∗^, and^∗∗∗^ implies statistically significant at 10%, 5%, and 1%, respectively. Source: Survey data, 2018. Ksh: Kenyan shillings, Robust SE: robust standard errors.

## Data Availability

The data used to support the findings of this study are available from the corresponding author upon request.
